# Challenges Facing Arab Researchers in Publishing Scientific Research: A Qualitative Interview Study

**DOI:** 10.21203/rs.3.rs-3129329/v1

**Published:** 2023-07-14

**Authors:** Alya Elgamri, Zeinab Mohammed, Karima El-Rhazi, Manal Shahrouri, Mamoun Ahram, Al-Mubarak Al-Abbas, Henry Silverman

**Affiliations:** University of Khartoum; Beni-Suef University; Sidi Mohamed Ben Abdellah University; University of Jordan; Aut Even Hospital; University of Maryland, Baltimore

**Keywords:** Arab region, scientific writing, publications, investigators, research literature

## Abstract

**Background:**

Studies have shown an underrepresentation of researchers from lower- and middle-income countries (LMICs) in the research literature compared with their counterparts in high-income countries. We aimed to explore Arab researchers’ challenges regarding conducting and publishing research in peer-reviewed journals.

**Methods:**

We used a descriptive qualitative study design of semi-structured in-depth interviews. Using purposive sampling, we recruited participants from four Arab countries in the Middle East and North Africa. All interviews were recorded, transcribed, and translated to English if the original language was Arabic or French. We analyzed the transcripts using reflexive thematic analysis. Several authors independently coded the transcripts and agreed on the identified codes, themes, and subthemes.

**Results:**

We performed 17 interviews: three from Egypt, six from Jordan, four from Morocco, and four from Sudan. Our participants’ comments were divided into three broad categories with associated themes and subthemes. The first regards the conduct of research (themes of inadequate quality of research, insufficient research resources, and nonsuppurative research environment). The second category involves the publishing process (themes of poor scientific writing skills and difficulties navigating the publishing and peer-reviewed system). The third regards international collaborations and the final category recommends methods to address the challenges. Our recommendations include: enhancing the institutional research culture, increasing funding mechanisms, establishing mentoring programs and workshops on research methodology and scientific writing, and increasing the representation of LMICs on the editorial staff.

**Conclusions:**

Identifying the challenges of Arab researchers in publishing original and quality research would guide programs tailored and targeted toward Arab scholars’ needs.

## Background

Disproportionate research outputs and unequal distribution of authorship among researchers from different world regions is a well-known observed phenomenon and serves as a barometer of research capacity and ownership [1]. The overall pattern from several studies reflects that while there has been considerable growth in health research in low- and middle-income countries (LMICs), there has not been a fundamental change in the global proportion of research production [2]. Essentially, disparities in output between researchers from LMICs and high-income countries (HICs) persist [3, 4]. The underrepresentation of researchers from LMICs in the literature compared with their counterparts in HICs has been demonstrated in several research fields, including maternal health, community health, surgery, infectious disease, and psychiatry [5]. These disparities are problematic, as locally produced research is essential to defining research priorities that are relevant to the context of the local health problems to ensure that research informs policy and practice [6, 7]. Challenges to conducting high-quality research include variability in access to mentors and funding opportunities and lack of support for professional development [8]. Structural barriers include inadequate resources, skilled personnel, and advanced laboratory facilities. Finally, an underdeveloped regulatory and ethical framework can delay research approval [9].

In addition to challenges from conducting high-quality research, many structural barriers prevent researchers in LMICs from publishing their research. Publishing in academic journals is essential to advance the careers of health researchers’ careers and to be competitive in obtaining grant funding. Several studies have investigated the challenges facing scientists from LMICs. For example, research writing instruction and support services are scarce in many LMICs, leading to poor manuscript preparation. One mixed-methods study showed that the main challenges to publishing a manuscript after presenting an abstract included time constraints, poor scientific writing or submission skills, lack of funding for publication fees, and results that are statistically nonsignificant [10]. Other studies have cited a lack of mentorship and sponsorship [11, 12], a lack of research infrastructure [13], and an unsupportive research culture as barriers to building successful research careers [14].

Even when LMICs researchers submit their manuscripts to journals, problems persist with the publishing process. Ehara and colleagues analyzed the reasons for rejection from international authors who submitted manuscripts to the American Journal of Roentgenology and found that the insufficient novelty of the research represented a significant issue [15]. These authors found a lack of new or valuable knowledge was the most common reason for rejection in all countries (44–76% of all rejections). Other factors that make publishing problematic are that many LMICs’ universities are not research-intensive and lack a critical mass of researchers and mentors [16].

Bibliometric studies focused on the trend of biomedical publications originating in Arab countries have shown a similar pattern of inequities in the published output. These have included biomedical fields such as HIV/AIDS [17], cancer studies [18], diabetes [19], integrative and complementary medicine [20], mental health [21, 22], ophthalmology [23], rheumatology [24], stroke [25], and substance abuse and toxicology [26]. While these studies indicate that the number of publications is increasing, they also show that Arab countries continue to contribute a decreasing percentage of the total publication output among other regions worldwide.

We are not aware, however, of any study that has investigated the research and publication challenges based on the voices of Arab scholars themselves. Thus, we aimed to explore Arab researchers’ challenges in conducting and publishing their research in peer-reviewed journals.

## Methods

### Study design:

We used a descriptive qualitative study design consisting of semi-structured interviews to explore the challenges faced by Arab researchers in conducting and publishing their research.

### Participants and recruitment:

We recruited participants from four Arab countries in the Middle East and North Africa: Jordan, Sudan, Egypt, and Morocco. We used purposive sampling. Inclusion criteria included researchers who have published research in English-language-based journals, are affiliated with public or private academic institutions, and specialize in biomedical or social sciences. All academic ranks were recruited from lecturer to professor. We contacted potential participants by email and phone.

### Procedure:

We performed semi-structured in-depth interviews. The interview guide was prepared from the literature review and the authors’ personal experience with challenges in publication. Supplemental 1 shows the interview guide.

Potential participants were allowed to perform the interview online using Zoom or face-to-face interviews. Before the interview, potential participants were informed in writing about the study objectives, methodology, and data protection. After obtaining consent, we conducted the interviews in English, Arabic, or French, per the participants’ choice. All interviews were recorded, transcribed verbatim, and then translated to English if the original language was Arabic or French. The number of interviews was determined by reaching data saturation.

### Data analysis:

We analyzed the transcripts using reflexive thematic analysis. The analysis was performed in six stages outlined by Braun and Clarke [27]. Data analysis began by engaging with the raw data. Several authors (AE, ZM, and KE) independently performed coding of the transcripts using MAXQDA software version 2020.

After the initial coding, the authors met together to reach a consensus on the coding. Subsequently, the authors explored themes and subthemes across the codes. After this stage, the suggested themes were revised with the data extracts. A second meeting with all authors was held to reach a consensus on the themes and subthemes.

### Trustworthiness:

To ensure the data’s trustworthiness, the authors fulfilled several of the criteria suggested by Guba and Lincoln [28]. The data analysis started after a prolonged engagement with the audio and transcripts. Member checking was performed by sending the study results to some research participants, and their comments were included in the final manuscript. Furthermore, the trial audit was granted a thick description of the process, good documentation, and preservation of the raw data.

## Results

We performed 17 interviews: three from Egypt, six from Jordan, four from Morocco, and four from Sudan. The average age of the participants was 43.6±7.7, nine were females, and eight were males; all participants had published research in English-based journals, and 15 had experienced manuscript rejection. Table 1 shows the themes and subthemes, which we will now present.

### Quality of Research

#### Inadequate novel research ideas:

According to participants, many studies were rejected by editors because the research lacked sufficient quality due to unsatisfactory novelty or failure to add new knowledge to the literature. For example, one respondent said:

“Arab researchers retake complicated topics that become old and solved. Therefore, it is common that many pieces of research made by Arab researchers are nothing, but the same topics of other research made by the European or Western Universities”.

(Interview 4 Jordan, Pos. 27)

Other participants said the following:

“However, we still tackle very old topics that humanity does not need anymore.”

(Interview 4 Jordan, Pos. 31)

“They either select the same previously handled topics or unimportant topics. For example, I have once received 17 pieces of research from a professor either for evaluation or promotion over the last two or three years. They were published either in noninternational English journals or Arabic journals. Each study is the repetition of another study with a different topic. I have rejected 13 pieces of research from the 17 ones. Their topics are not worth researching.

(Interview 3 Jordan, Pos. 137)

In response to an interviewer asking what the reasons for journal ejection are, one of the participants said:

“They [editors] mainly justify their rejection that there is much available data on the same topic and there are no new findings we provide in this research

(Interview 1 Egypt, Pos. 76–77)

#### Lack of access to the literature:

When asked about challenges for Arab researchers when literature does not exist or there is a lack of access to the literature, one respondent replied:

“The lack of literature might be a… starting point. It helps you highlight the gap and the need for your research. [However], we have limitations in locating the literature in the fact that the university does not give you wide access, for instance, to journals or publications…. In Jordan, paramount importance is given to gray literature, i.e., unpublished research. We have plenty. This is a significant source”.

(Interview 1 Jordan, Pos. 92)

#### Failure to critically analyze the literature:

Participants mentioned that the inability to conduct novel research is due to deficient critical analysis skills necessary to develop innovative ideas. One participant said:

“To be honest… challenges start from the idea’s inception to come up with innovative ideas. Do you know why? Neither analytical thinking nor critical thinking is used in our schools or universities. Our professional education or degrees do not teach us critical thinking in research”.

(Interview 5 Jordan, Pos. 27)

Another participant remarked that researchers do not recognize the opportunity to generate new ideas from existing research:

“Definitely, it is not required that everybody open a new topic of research. It is good to have several research on the same topic from different angles and perspectives in different demographics. Repetition is useful in scientific research. Simulating the same study with the same methodology and the same instruments, but on a slightly different population, at a slightly different time, or at a different geographic location is good and necessary. However, few researchers study new concepts, and others follow them”.

(Interview 2 Jordan, Pos. 122)

#### Flawed research methodology:

Several participants believed that poor choices regarding research methodology are one of the leading causes for the low number of publications from Arab researchers.

“The quality of some of our scientific production is less than desired because of some choices of methodology and the purpose of the study. Their justification is the lack of time and lack of resources”.

(Interview 2 Jordan, Pos. 91)

### Inadequate Research Resources

#### Lack of Funding:

Lack of funds was a frequently mentioned organizational challenge. Participants discussed the lack of resources in their organizations and its effect on publication. From the participant’s perspective, funding is needed to conduct high-quality research with a better chance of publication. For example:

“I think that if we have funds, they should give these $2000 to the researcher to fund his research requirements. Hence, he can do high-quality research that can be published in subscription journals”.

(Interview 1 Egypt, Pos. 11)

Another participant mentioned that insufficient funds is a leading cause of of low-quality research and a hindrance to performing randomized clinical trials :

“I would say that the reason most frequently given was lack of originality in the projects. That is, among other reasons, given our means, we have very few resources, so we tend to have retrospective or cross-sectional studies. We have very few prospective studies and even fewer clinical trials. We hardly have any clinical trials despite knowing that in our field, it is essentially accepted by prestigious journals.

(Interview 2 Morocco, Pos. 37)

Funding is also needed to pay the submission fee for high-quality, open-access journals. One participant said:

‘The problem with the funding budget for publishing is its costly fees”.

(Interview 1 (Morocco: 4)

#### Inadequate research facilities:

Participants mentioned that a lack of sufficient and advanced laboratory equipment will limit the nature of the studies that could be conducted or negatively affect the quality of the research results. For example:

“And this is one of the challenges that faced me personally. Therefore, when I was doing my research, I first targeted a journal that is very specialized in the research topic. After six months of submission, they rejected it because they said simply, that I did not do a very tiny laboratory test that I could have done if I had the facilities in the lab. Therefore, this could be a real challenge”.

(Interview 2 Sudan: 64)

Another participant said:

I know researchers with very old equipment but need spare parts or reacting agents. If the same researcher works on the same research outside Sudan, there is a huge difference in productivity.

(Interview 3 Sudan, Pos. 40)

#### Lack of statistical resources:

Participants mentioned the lack of resources for performing statistical analyses. One participant said:

“If you have a research department composed of biostat, and you will have your own [biostatistician] that will be available for you at any time…this will add a lot to the value of your research,

(Interview 1 Sudan, Pos. 145)

As explained by some participants, the lack of electronic databases and electronic patient records was also considered a challenge and a limitation in producing high-quality studies. They mentioned that sometimes reviewers and journal editors would ask investigators to verify their presented data. This would be di cult to verify as the data would not be available electronically.

“We do not have a data collection which is electronic base. Therefore, we always use the manual one, this will be very di cult.”

(Interview 1 Sudan, Pos. 23)

#### Inadequate library resources:

Several participants mentioned the lack of library resources as hindering manuscript writing and conducting research. One participant said:

“Yes, it is really a major challenge. Through Hinari and others are closed. Sometimes, we had to pay to get open access by paper. There was one young patient with a metastatic lung tumor. I looked for articles regarding tumor metastasis. I must pay to access the papers that reviewed the older papers. This is universal. All our Arab countries suffer from financial support”.

(Interview 5, Jordan, Pos. 57)

The interviewer asked the participant why Arab researchers open access to journals and the response do not have was as follows:

“The institutions must pay. As you know, the national library should be the resource of knowledge. However, it must provide open access.”

(Interview 5 Jordan, Pos. 57–59)

Another participant highlighted the benefits of having free access to the literature:

“The library of the Supreme Council of Universities…provides all the previous research in terms of abstract and titles. It prevents the repetition of a paper and plagiarism. It motivates communication. For example, I can know the contributions of other universities from other cities. Therefore, I can contact the researchers to learn more about the methodologies. Therefore, I accumulate with my research to his results. This did happen with several colleagues. Moreover, it can be accessed by any mobile device.

(Interview 2 Egypt, Pos. 52)

### Nonsupportive institutional research environment

#### Inadequate protected time to conduct research:

Participants mentioned that balancing teaching responsibilities and research activities in academic institutions is extremely difficult. Several participants agreed that competing academic obligations were one of the significant personal challenges. One participant said:

“The professor gets frustrated by his huge managerial and teaching responsibilities. He should also serve the community. Our responsibility is to teach students, supervise the masters, serve society, prepare the Ph.D., and do managerial tasks as the decider of one of the faculty committees, the dean’s assistant in a certain task, the manager of a department, and so on. All of this is time-consuming, frustrating, and energy consuming.”

(Interview 2 Jordan, Pos. 87)

The lack of time was also associated with financial difficulties. This was explained by the fact that most of the researchers are affiliated with universities and academic institutions; according to participants, salary is meager at these institutions, which will cause the researcher to look for another supplementary job, adding additional workload and time constraints. For example:

The governmental salaries of a researcher are minimal. Therefore, he should work a second job in the private sector for his living. Therefore, he does not have sufficient time to perform scientific research for the academic institution with which he is affiliated.

(Interview 2 Egypt, Pos. 6)

#### No Rewards for research activity:

Several participants mentioned that their university demotivates them regarding publication because of the lack of rewards. For example:

“It is very human that we are rewarded for what we do and when we are not productive, we do not get any rewards. Therefore, publishing is a hard job. It involves a lot of evening hours; it is very di cult to be committed to publishing unless one gets something out of it”.

(Interview 2 Sudan: 68)

Several participants mentioned the need to support researchers with rewards and incentives after publication.

“Researchers should be appreciated and financially rewarded for their contribution to the university’s global reputation. This reward is considered an incentive for them”.

(Interview 2 Jordan, Pos. 192)

#### Bureaucratic hurdles:

Participants mentioned institutional bureaucracy as a barrier hindering researchers’ publications. One participant gave an example of the IRB delaying its review to the degree that the research might no longer be novel or unique. The participant said:

“By the time I have an innovative idea, then you go here and there many times… then [I fill in] the IRB form, so the research proposal can be reviewed, and then I submit for funding. Therefore, by the time when people [researchers] are ready [to work on it], the idea gets old. I have experience with a paper that I wrote. Nobody had written about it. By the time we finished it, I found thousands of researchers who had written about it. What can I do more? That is why we go to the second-class of journals”.

(Interview 5 Jordan, Pos. 71)

#### Perverse requirements for promotion:

All participants heavily criticized the academic promotion regulations at their institutions. According to participants, most institutions have regulations that govern promotion from one academic rank to another based on the number of publications, not their quality. Participants believe such regulations encourage researchers to look at quantity, not quality.

“The problem is that we look for quantity rather than quality. Maybe they are looking for both, but the universities want to be proud of the number of research that their students have done. There is a problem here with linking career promotions to the number of research [articles]. Such research has certain conditions that help those who put quantity over quality. Therefore, we have a general culture that encourages researchers to care for quantity more than quality”.

(Interview 6 Jordan, Pos. 59)

Another participant said:

“The researcher should be evaluated by the research activities and effort and not only by the publication number. The special pathway that aims at academic career promotion should consider the efforts of the researchers instead of the publications. Not necessarily to get the full score like the publication, but at least if the researcher is conducting research, they encourage and monitor him. Therefore, I think that universities should consider that”.

(Interview 6 Jordan, Pos. 63)

Participants reported that when the primary motivation driving Arab researchers to publish research is to obtain a promotion, it hinders intrinsic motivation to generate original knowledge. Although it is not wrong to pursue a promotion, such motivation, they believe, is not enough to pursue research to produce more knowledge and publish more research. This was explained by one participant:

“Many researchers’ purposes is not the research itself, but their purpose is different either achieving job promotion or other goals.”

(Interview 3 Jordan: 135)

When asked whether “the desire of the researcher can affect publication and that the lack of desire may be a challenge to publish? One participant said:

“Yes, definitely, it will affect the research process. If you love something, you will do it, you become obsessed with it.

(Interview 1 Sudan, Pos. 206–209)

#### Lack of Mentoring:

Participants mentioned the lack of mentoring as another challenge to publish. They think the publication process needs skills that a mentor should teach at the beginning. For example:

“I remember when I was a dental student, a dental student, and I was doing, um, my undergraduate research, I told my supervisor about publishing it. In addition, yet he said to me, yes, yes, you can do that, but he did not give me any guidance, and at that time, he was too ignorant, so basically, if I had a supervisor who has, um, experience in publishing, I could have published when I was a student. Therefore, I think this is a very critical thing during undergraduate life”.

(Interview 2 Sudan, Pos. 160)

#### Insufficient Training:

Many of the participants agreed on the importance of training and that their institutions should invest in training at different levels, beginning with including undergraduate education:

“I think undergraduate is a very good starting point when they are they when they are given assignments, and these assignments are properly assessed for them. This will help them, um, learn how to do um, scientific writing, and eventually how to publish their work”.

(Interview 2 Sudan, Pos. 160)

Training should be continued throughout the researcher’s journey; one participant stated that even professors need training.

“The more important is to provide training as an academic entity. There should be training for professors, for example, on how to publish in SCOPUS and the benefits of publishing in SCOPUS. The university must provide the guidance, recommending some journals to them, send periodically some high-ranked journals”.

(Interview 4 Jordan, Pos. 131)

#### Nonsupportive research culture:

Participants mentioned that the general research culture at the institutions is sometimes not supportive of research, as it does not encourage or prepare young researchers for this activity.

“Our culture did not care for research. By ‘caring’, I do not mean that the professor requests his students to do research; I mean that the culture of designing scientific research is not proper. When students start to engage in higher studies, they begin to face unexpected challenges…. It takes a lot of time due to the challenges”.

(Interview 6 Jordan, Pos. 42)

One researcher compared his experience in two universities, one in Australia and the other in his home country; he said:

“At the University of Melbourne where I did my higher studies, the difference is that Australia provides an environment that encourages research. Most of my research was clinical, so I needed patients. It is normal there for everybody to help researchers in doing their research by transferring the patients who could contribute to the research and that conform to the conclusion criteria. This is because everybody there realizes the importance of research. Everybody behaves as if it is a culture there. Other environments suffer due to lack of this culture”.

(Interview 6 Jordan: 44)

Another participant said:

Therefore, he was speaking about the general culture of the place they are working in. If it is promoting publication or not, if they are giving them the time, giving them space, giving them what they need to publish, they will publish.

(Interview 1 Sudan, Pos. 202)

When asked whether the environment was supportive enough for scientific research, the participant said, “It is not a supportive environment.” (Interview 2 Egypt, Pos. 33–34). Another participant said the following:

“Unfortunately, the research environment, in general, suffers from the problem of integrity like plagiarism or people not contributing to the work and everyone takes credits.”

(Interview 2 Egypt, Pos. 38–39)

In contrast, another participant described the experience of a supportive culture:

“First thing, because it is a university, all people there surrounding you are always in an academic state of publication, research, and questions. Therefore, it is a very supportive environment that helps you to overcome a challenge…. This motivates us. It is a highly motivating university, except the pressure of work exists, especially in the first two semesters…. I adapted to the new environment and people. Then, I feel stability. I would like to continue researching.”

(Interview 3 Sudan Pos. 39–40).

#### Deficient Teamwork Culture:

Several participants highlighted difficulties associated with assembling a research team and its effect on the conduct of high-quality research. Some participants think they usually lack the required commitment even if a research team is working on a project. For example:

“I think the main obstacle that I faced when working with my local peers was the lack of organization. Therefore, we do not commit to our plans, even if we plan something in a timely matter; for example, to complete this section within this time, we do not commit to it. In addition, I would be honest with that, even me myself, I do not commit to things when I work with my local peers. For some reason, it is like the culture.”

(Interview 2 Sudan, Pos. 132)

Continuing this theme of culture, another participant mentioned the lack of a culture for teamwork and said:

“In management science, we study how to form teams, and how to manage its production. Teamwork in the Arab world represents an issue maybe because of the shortage of the teamwork culture to manage the teams for achieving the tasks.”

(Interview 4 Jordan, Pos. 55)

Other participants said the following:

“It is rooted in the culture. We did not learn to be in teamwork. Everybody wants to work secretly.”

(Interview 4 Egypt, Pos. 123)

“We have not acquired the spirit of teamwork. The culture of teamwork and collaboration needs to be motivated. We are used to playing his role alone. Therefore, the researcher registers his study alone, works on it alone, and succeeds alone. This needs to be motivated.”

(Interview 2 Egypt, Pos. 66)

When asked whether there is a difference between the concept of a team at highly ranked Western universities and Arab universities, the participant said:

“The only difference outside Sudan is that the rules are much strict [regarding authorship], but in Sudan, I could engage in a project and still work on it, and then I am surprised by my work getting published under the author’s name of one of my colleagues…. Unfortunately, the research environment, in general, suffers from the problem of integrity, such as plagiarism or people not contributing to the work, and everyone takes credit. Rules in Sudan could be stricter in terms of the evaluation of individual work.

(Interview 3 Sudan, Pos. 37–38)

### Difficulties with Scientific Writing

#### Language barrier:

A frequent challenge mentioned by the participants included the language barrier. Participants reported that writing in English is one of the significant difficulties they face regarding scientific publication, as it is their second and sometimes third language. One participant mentioned:

“Many Arab researchers suffer from deficiencies in the English language. This hinders them from publishing in highly indexed journals with international influences. Therefore, the English language is an essential obstacle. It is also an obstacle to follow the information of the most recent research of the advanced universities that are written in English”.

(Interview 4 Jordan, Pos. 31)

However, one participant believed that this should not be considered an unsolvable problem, as other nonnative English-speaking countries had crossed this barrier; she explains:

“Other countries, like China, for example, do not even sound in English, even in undergraduate, but they have professional companies and professional, scientific writers. Therefore, you can just give them your article in Chinese. In addition, they formulated for you in a very sound scientific English. In addition, if you notice, there are a lot of publications coming from China, and in English”.

(Interview 2 Sudan, Pos. 36)

#### Poor scientific writing skills:

Another participant explained that it is not just English proficiency but instead being able to write in a scientific style.

“You could excel in the English language in terms of grammar and vocabulary, but how to write scientifically. How to write it? How to divide your scientific writing. For example, what should I have in the introduction? What are the sections of the discussion part? I have read papers as a public case”.

(Interview 5 Jordan, Pos. 27)

#### Inadequate publication services:

Several participants mentioned the need for publication services. They believe organizations should provide editing, proofreading, and plagiarism-checking services. One participant said:

“There should be an office to answer their questions about the publication. There should be a research publication assistant. He should assist you in publishing your papers.

(Interview 3 Sudan, Pos. 136)

Another participant mentioned the high cost of editing services to help with poor scientific writing skills:

“Yes, of course. As our mother language is not English, we suffer from many linguistic issues. We also suffer from problems in scientific writing. However, this is not a problem limited to Arabs. For example, it is rare in Japan to find a person with good English. Most Japanese researchers send their papers to English proofreaders…The editing cost is very high. [The Japanese] write their papers in the Japanese language, and they send them to a company to rewrite them in English. Then, this company would send this English paper to another English company to edit the English language of the paper. Hence, the cost could increase by $1500-$2000”.

(Interview 1 Egypt, Pos. 91)

Participants mentioned that the organization should provide funds to support scientific publications. Funds should be regulated closely and should be spent carefully.

“There should be real financial support to scientific research with control and laws so that there’s no corruption. Everything should be controlled”.

(Interview 2 Jordan, Pos. 190)

#### Plagiarism:

Participants mentioned plagiarism as a challenge. One participant said the following:

In Sudan… we were not trained during our undergraduate years to write scientifically and free of plagiarism…. In addition, one of the things that I believe helped me very much in publishing my article is that I did my masters in the UK, and plagiarism and scientific writing were an essential part of our training there. Therefore, I could access the software to help me know what is the percentage of things that I am taking from others without citation, and this way, I could avoid plagiarism. Because sometimes, when you publish, you get your paper rejected because they find plagiarism, and in scientific publishing, there is zero tolerance for plagiarism.

(Interview 2 Sudan36–46)

#### Publication Submission Process:

Several aspects of the publication process proved to be problematic. These include the following aspects:

#### Choosing the right journal:

Journal selection is an essential process that can hinder publication if a manuscript does not fit within the journal’s scope. Several participants said the following:

“The challenge I am currently facing is how to choose the journal? This is my biggest problem”.

(Interview 3 Sudan: 40)

“And this is one of the challenges that faced me personally. Therefore, when I was doing my research, I first targeted a journal that is very specialized in the research topic. Furthermore, they rejected my work.

(Interview 2 Sudan: 64)

However, another participant explained how he/she selects the right journal:

“After I started reviewing the manuscript, I explored the journals that are suitable for my topic. Oral pathology research is limited because I care for the journal criteria with good impact factors and the minimum rejection rate. Therefore, I kept reading the requirements of the journals before I started.”

(Interview 3 Sudan, Pos. 35–38)

Nonetheless, several participants mentioned that the difficulty with choosing the correct journal partially emanates from many Western journals not being interested in topics essential to LMICs. One participant said:

“The most di cult problems that we face [with] the international journal…is that they serve certain goals and perspectives…. Such standards are defined based on …Western culture. This defines the kinds of topics that they accept. Our local topics are totally different from theirs…There is a big problem with the topics that are important for them”.

(Interview 3 Jordan, Pos. 38)

#### Authorship Disputes:

Authorship issues were discussed by many of the participants and their negative effects on publication. One participant explained that some researchers prefer not to publish their work since their supervisors would insist on placing themselves as first authors, although they did not actively contribute to the work:

“I hear some of my colleagues are not interested in publishing their research because they know that even if they did most the most work, their supervisor would still be the first author because the supervisor just wants to be the first author. In addition, they cannot do anything about it. Because their supervisor would make the problems, you know, these types of things”.

(Interview 2 Sudan: 146)

Others explained that the need to include guest, ghost, or gift authorship in publications is a prevalent issue. They find this very depressing and unfair to those who did the work. Such adverse effects demotivate researchers from publication.

“Just by the simple fact that he is the head of the department, we must include him.”

(Interview 4 Morocco, Pos. 228)

#### Difficulties writing the cover letter:

Writing a convincing cover letter was mentioned as another obstacle in the submission process. One participant said:

“How to formulate a cover letter, which is a very important step in your application process, you need to write something that will grab the attention of the editor. Sometimes your manuscript will get rejected only at the editor level because your cover letter is simply rubbish, compared to today’s standards”.

(Interview 2 Sudan: 32)

#### Unhelpful and callous reviewers’ comments:

Receiving reviewers’ comments was emotionally draining after rejection. Some participants reflected on their experiences where they felt the comments were out of context, ill-informed, aggressive, or depressing.

“They managed to get me a bad feeling because, or maybe because I could do not think about what they asked me.”

(Interview 2 Sudan, Pos. 86)

Other participants explained that reviewers sometimes make unwarranted comments that delay the publication process. For example, one participant said:

“And it has happened to me sometimes, many times, to submit articles and we have found that the reviewers do not master some statistical aspects. For instance, they make comments that are out of the context, and it delays the publication”.

(Interview 4 Morocco, Pos. 55–57)

#### Responding to reviewers’ comments:

Participants raised the issue of writing effective responses to reviewers’ comments, which represents a critical step that requires special skills. One participant explained the following:

“I would say responding to reviewers’ comments is one skill that you need to have when publishing. Because you cannot just agree with every reviewer’s comment because some of them are not valid. Therefore, you need to analyze what they are saying, agree with some and disagree with some.”

(Interview 2 Sudan: 120)

#### Occult Discrimination:

Several participants raised the issue of subtle discrimination, although it might not be intentional. One participant explained:

“For me, this is discrimination… I think there is a conscious or unconscious orientation to favor or give an advantage to the works of teams from Western countries. That is my opinion”.

(Interview 4 Morocco, Pos. 177)

Another participant believed that some editors might reject high-quality research from the Arab region because it will not be cited frequently. Moreover, researchers and editors from other regions perceive manuscripts from the Arab regions as low quality.

“Because of perception of poor quality, the editors reject the papers from the region, [also] these papers are not cited in other research, which would impact the journal’s impact factor…When our papers are published in journals, they are usually not cited since the other researchers who can cite our papers question the quality of the science in our papers. Therefore, they do not get cited. They speak about the perceived quality”.

(Interview 5 Jordan, Pos. 81)

However, other participants believed that the author’s origin does not affect the editor and reviewers’ decision in publication.

“It is not something personal; it is something related to the infrastructure, it is not just because you are from a developing country. I believe it is not like this, but you are in a developing country; you have limited resources so you will limit your way of research”.

(Interview 1 Sudan: 168–169)

One participant shared an interesting thought, as he believes that Arab researchers should be more interested in participating in editorial boards; from his point of view, that will help increase publication from the Arab region.

“We – the Arab researchers - should be more engaged in the editorial boards of the journals since we are not represented there. If you have a look at a prestigious journal, it is rare to find an Arab person on its editorial board. Most of them are either Americans or Europeans. Sometimes, it includes Chinese, Koreans, Indians, Russians, and even South Africans, but not Arabs. This could impact our rights.

(Interview 2 Jordan, Pos. 190)

### International collaboration:

Many participants mentioned the value of international collaborations as an effective way to enhance research activity and increase publications. Collaboration could take the form of a research team from different countries. Participants believe such collaborations can solve issues regarding the lack of funding and research resources and provide other tangible aspects.

#### Enhance Commitment:

Participants believed that the opportunity to work with international collaborators would provide them to adopt a substantial commitment toward research tasks greater than working in their local institutions. One participant said:

“If an international institute invites me, this could be better, and you commit. The more formal the relationships are, the more commitment there is to the research.”

(Interview 3, Sudan, Pos. 107)

This difference in commitment level in research is thought to be due to appreciation of the outcomes, as explained:

“If you look at the roots of the main difference between local and international research teams, it is because international groups would appreciate their work, but we do not feel the benefit that could come from our hard work”.

(Interview 2 Sudan, Pos. 138)

#### Improve quality of research:

International collaboration can also enhance the quality of research. For example, by adding different populations and increasing the impact of the result and by providing the required technical support that can improve the quality of the research, as explained by one participant:

“If you have a questionnaire, you will have another environment, and you are not limiting your research to your environment or culture; the publisher is always looking for something that can apply to everyone. Therefore, this adds value to research. The second is that sometimes you want to conduct the research, but you do not have the infrastructures that can support you. Therefore, if you make an international collaboration…, you can have help from other countries to help conduct the research”.

(Interview 1 Sudan, Pos. 155–157)

Participants also emphasize the sharing of ideas. When asked, “How do you differentiate between the work within an Arab researcher’s team and an international researcher’s team in terms, quality, discussion, publication, and so on,” the participant said,

“The difference is in the sharing of ideas. Discussing with integrated researchers who have already published many papers is more valuable than discussion with others. They are not necessarily from Western countries. They could be from our Arab countries. Their experience is unique and meaningful”

(Interview 6 Jordan, Pos. 80–81)

Another participant described the advantages of working with international researchers:

“It is more beneficial, and there is lots of sharing: sharing tasks, sharing knowledge. It was good. We do not have this culture of sharing and teamwork.”

(Interview 1 Morocco, Pos. 81–86)

When asked whether it was easier or more challenging to work with international collaborators compared with local collaborators, one participant said:

“So, based on my experience, the local collaborators are involved mainly in giving data but not in the process of writing, the methodology and the design of the study, nor in the process of drafting the article, submitting it, and reviewing [it] with readers’ remarks, etc. Therefore, I this type of collaboration limits the desire to do multidisciplinary work. Whereas when working with international researchers, we often have parts that are dedicated to this author, a second part dedicated for the writing to another author, etc. Therefore, we have a more responsive collaboration.”

(Interview 2 Morocco, Pos. 75–76)

#### Enhance Scientific Writing:

Participants also believed that international collaborations could help improve scientific writing style, English proficiency, and having well-known figures in the field as collaborators, which will be reflected positively on any grant application, increase the project’s trustworthiness, and solve many fund-related challenges. One participant said:

“Some of us were fortunate enough to get our Ph.D. in Western countries. So, they learned Western academic writing. They were exposed to American or European [who are] the best as advisors and professors who are scientists in our specialty. We learned from them how to write successfully. Sometimes, they cooperate with us as co-authors in some of our scientific productions. This increased our chances of getting published in prestigious journals”.

(Interview 2 Jordan, Pos. 58)

### Addressing the challenges

In addition to pursuing international collaborations, participants gave further suggestions for addressing the challenges of conducting quality research and achieving publications in high-impact journals. These suggestions included training in the different research methodologies, enhanced teamwork culture that could increase efficiency and provide motivation, increased funding mechanisms, and having individuals from the Arab countries serve on the journals’ editorial boards. Examples of representative quotes include the following:

“Another point is the training. It should include the new methods…. I submitted all my papers in the quantitative method. Even within the quantitative, there are several methods.”

(Interview 3, Jordan, Pos. 231)

“…the more you have researchers engaged in the paper, the more everyone is motivated. I have succeeded in getting my two studies published since my coresearcher was active. He always encourages us throughout the research period and guides us to do it.”

(Interview 3, Sudan, Pos. 62)

“Another important factor is the lack of real money for scientific research. I once requested 7000 Jordanian dinars for a scientific project, but they gave me only 4500, so I could not buy a printer for my research project when I truly needed one. Fortunately, I had another one in my office. There should be real financial support for scientific research with control and laws so that there is no corruption. Everything should be controlled.”

(Interview 2, Jordan, Pos. 190)

“The Arab researchers should be more engaged in the editorial boards of the journals since we are not represented there. If you have a look at a prestigious journal, it is rare to find a Jordanian or an Arab person on its editorial board. Most of them are either Americans or Europeans. Sometimes, it includes Chinese, Koreans, Indians, Russians, and even South Africans, but not Arabs. This could impact our rights.

(Interview 2, Jordan, Pos. 190)

## Discussion

Our study reports on the challenges of Arab researchers regarding conducting and publishing their research. While several studies have examined the research and publication challenges regarding similar issues, this is the first study exploring the voices of Arab researchers regarding the specifics of such challenges from their viewpoints.

Our participants’ comments can be divided into three broad categories with associated themes and subthemes. The first regards the conduct of research (and associated themes of inadequate quality of research, insufficient research resources, and nonsupportive research environment). The second involves the publishing process (and the associated themes of insufficient scientific writing skills and difficulties navigating the publishing and peer-reviewed system). The final broad category regards international collaborations, which can address the preceding two categories.

Our results confirm findings from other studies. For example, Majid and colleagues performed a mixed-methods study to determine the barriers to publishing full manuscripts by postgraduate trainees from Pakistan [10]. Their survey showed that the main challenges included lack of time, limited scientific writing and submission skills, lack of funding for publication fees, statistically nonsignificant results, and low priority for publication in their research culture.

Additionally, Weathers et al. reported that the main barriers to publication for postgraduate year-one pharmacy residents at the University of Utah, USA, included lack of time, insufficient knowledge of the publication process, lack of research mentorship, substandard quality of the study that is not suitable for publication, and delays in obtaining approval from the respective institutional review boards [29].

We will now discuss many of these challenges from our significant categories revealed by our Arabic participants.

## Performing quality research

### Novel Research:

Many of our participants mentioned their inability to generate novel research suitable for publication. Their challenges compared with Ehara and Takahashi, who analyzed rejections from international authors who submitted manuscripts to the American Journal of Roentgenology. They found that lacking new or valuable knowledge was the most common reason for rejection in all countries (44–76% of all rejections) [15]. Scientific merit, including the importance of the topic and whether the research adds to the existing knowledge base, represents the prime determinant of the quality of a scientific publication. Furthermore, from this study, investigators from countries where English is the primary language had higher acceptance rates than those in which English is not the primary language (29.1% vs 40.3%, p < 0.05). These authors commented that while errors in research methodology and data analysis, poor language, and errors in a manuscript organization are flaws that can be correctable to some extent, choosing a research topic that lacks originality cannot be salvaged. This issue highlights the importance of a complete literature review to explore the existing research gaps to identify options for choosing an innovative research topic [30, 31].

This issue of novelty begs the question of what counts as innovative research. At first glance, the rejection is because the research topic falls out of the journal’s scope. Determining the journal’s scope is often related to the specialty of the journal. However, in many cases, Arab researchers conduct research investigating illnesses within their health context, often not relevant to American or European journals [32]. Researchers in other LMICs face similar issues in justifying the relevance of research that addresses their context. Hence, to get published in high-impact journals, LMIC researchers need to conduct research relevant to HICs, which will further enhance the health inequities between HICs and LMICs.

### Deficient Research Resources:

Funding for LMICs research is deficient due to resource constraints and the failure of LMICs’ governments to allocate meaningful resources for research and capacity-strengthening for academic and research institutions [16]. Hamdeh and colleagues examined trends in cancer research in the Arab region and identified that Arab investigators performed marginally relevant research to improve cancer health in their populations [18]. In contrast, HIC governments find value in generating high-quality evidence to inform policy and practice.

### Institutional Research Culture

Several aspects related to the research culture in Arab institutions inhibit research productivity. For example, due to the limited options for a research career, potential researchers might only implement research projects as a requirement for partial fulfillment of their degrees and not as a pathway to a defined career trajectory. Our participants cited other obstacles, including lack of funding, insufficient training, paucity of resources for training and scientific writing, and inadequate research infrastructures. Other inhibitory aspects of the institutional research culture include the following:

#### Promotion Policies:

Academic institutions embrace discouraging promotion policies emphasizing the quantity of research rather than quality. Several of our participants believed that the lack of rewards at their universities demotivates investigators from conducting research with subsequent publications.

#### Protected time:

The lack of advancement of successful academic research careers for junior investigators is usually due to two commonly reported challenges: (1) insufficient time to focus on research and (2) conflicting work expectations, including heavy teaching or clinical loads while pursuing research.

#### Insufficient Mentoring:

Graduates pursuing research careers are inadequately prepared and often require intensive mentoring by senior researchers. Ineffective mentorship from senior investigators in graduate schools weakens the foundation to build their academic careers. Many of the LMICs’ universities are not research-intensive and lack a critical mass of researchers and mentors grounded in research [26]. Jatin and colleagues performed a focus group discussion study and showed that many junior researchers look to mentors for support and reassurance and to play a significant role in guiding them [33].

#### Teamwork:

Our participants mentioned that a culture emphasizing teamwork is lacking in their institutions; many investigators prefer to work alone. Group support has been shown to increase the frequency of publications by emphasizing the group process and respectful collaborations [34].

### Navigating the publishing process

In addition to barriers to conducting research, Arab investigators face challenges with getting published in high-impact journals. LMIC researchers might be underrepresented in scientific journals because of the dominance of English in the research literature, power dynamics that determine the order of authorships, and the perceived or actual bias of editors toward Western researchers [35, 36]. Other challenges include the following:

#### Scientific writing:

Many participants emphasized deficiencies in scientific writing that present publishing challenges. Research writing instruction and support services are unavailable in many low-resource settings, leading to poor manuscript preparation. In addition, academic journals lack the staff and budgets to offer extensive writing support services to authors who submit promising but poorly prepared manuscripts [5]. Furthermore, international English journals adhere to specific writing style standards, favoring manuscripts written by native English speakers. Editors and reviewers reject manuscripts with promising results if they lack writing development. Finally, Shah and colleagues found from their interview study that “cognitive burden” was a significant inhibiting factor in the writing process, as participants considered the task excessively complex and demanding [33].

The challenges in scientific writing make it critical to identify the characteristics of publishing interventions for researchers in LMICs that would result in the most meaningful gains in technical writing skills and publications [5]. Busse and colleagues performed a systematic review to evaluate interventions for instruction in writing and publishing [3]. Across interventions, those that stressed the importance of a high ratio of mentors to participants, the need to accommodate the time demands of busy researchers, and the necessity of a budget to support open access fees and high-quality internet connectivity proved most successful.

Finally, commentators have shown the value of group writing. For example, Galligan et al. demonstrated that collegiality was strongly apparent in all writing groups [37]. Furthermore, in their interview study, Shah and colleagues found that participants favored the idea of group writing. The responses emphasized the role of colleagues, friends, and mentors in aiding the writing process [33]. Finally, Pololi et al. demonstrated that participants working in pairs produce shorter but better texts regarding grammatical accuracy and complexity [38].

#### Publication process:

Our participants mentioned several conventions in scientific publishing that present further challenges to researchers. These include the importance of writing a cover letter to accompany a journal submission and the need to consult a journal’s author guidelines throughout the writing and formatting process; access to scientific mentors would be helpful to navigate these aspects of the publication process. Pre-Publication Support Service (PREPSS) is a nonprofit, nongovernmental organization that works to meet these needs. PREPSS provides onsite training, peer-review, and copy-editing services to researchers in LMICs who wish to publish their health research in peer-reviewed journals. The PREPSS model is one of many interventions necessary to restructure global health research to better support health researchers in LMICs and reduce current power imbalances. [5].

#### Authorship disputes:

Many participants discussed challenges in determining authorship rank as hindering publication. A not uncommon practice is that mentors are gifted first authors even if they have not contributed to the research. Revera discussed authorship malpractice, including “authorship disputes, excessive coauthorship, monetary incentives, authorship issues in student-mentor relationships” [39]. There are also unfair authorship practices in North‒South collaborations due to power differences between researchers from HICs and LMICs and real or perceived bias in medical journals that favors prominent Western researchers as first authors [35, 40].

### International Collaborations

Many participants endorsed collaborations with international investigators as an efficient method to address barriers and increase the likelihood of quality research and subsequent publications. A diverse research workforce is advantageous because the diversity of viewpoints leads to a wider variety of novel research ideas for studying emerging diseases, treatment responses, novel clinical decision-making tools, and ways to reduce health disparities [8]. Furthermore, LMIC universities collaborate with institutions from HICs to obtain funding.

Despite our participants’ advocacy for international collaboration, problems exist. For example, power dynamics inhibit fair international collaborations between researchers from HICs and LMICs [5, 41]. Other significant issues involve the fairness of authorship rank and the sharing of funding [35]. For example, one study showed that whereas the absolute number of LMIC first authors has increased, it has declined as a proportion of all authors. The relative rate increase in first authorships post-2000 was 11.8-fold for non-LMIC first authors and 2.8-fold for LMIC authors. LMIC first authorship increased over time for research funded by LMIC, but LMIC’s first authorship declined over time for research funded by high-income countries (HICs) [4]. Additionally, in studies where authors from LMICs and HICs collaborate, authors from LMICs are less likely to be represented in the first and last author positions, which reflects disparities in leadership and decision-making power [3]. This reduced participation among researchers in LMICs is further revealed by their limited presence as corresponding authors [41]. The disparity between LMIC and HIC first authors can be partially rectified if international funders develop mechanisms that direct funding to LMIC researchers, which can lead to greater equity in global health research [42][1].

### Recommendations

Based on the voices of our Arabic participants, we offer several recommendations that could address several of the major challenges to conducting and publishing research.

#### Enhance Institutional Research Culture:

There is a need to inculcate a research culture at LMIC institutions to encourage junior investigators to perform research and publish their research findings, as these trainees will become future researchers. Institutions can promote such a culture by requiring peer-reviewed international publications for promotion, establishing a culture of teamwork to enhance productivity, providing protected time, and developing promotion guidelines that reward quality research.

#### Mentoring programs:

Mentoring programs should be developed by pairing junior researchers with senior researchers who can guide project research development and execution, and manuscript writing.

#### Funding:

Although research funding is very competitive, funders have a great deal of leverage to shift practice by changing application requirements that include research collaborations to maximize equity between researchers from HICs and LMICs [42].

#### Workshops/symposia:

Institutions should provide workshops on scientific writing and the publication process. Such workshops for trainees of LMICs are needed to enhance their writing and time management skills and help them navigate the steps involved in the publication, e.g., selecting the proper journal, writing a cover letter, and responding to reviewers. Institutions should also offer services that include editing, proofreading, checking for plagiarism, and financial support for research and publication services.

#### Increased representation of LMICs on editorial staff:

Several participants mentioned the lack of Arabic representation of journals’ editorial boards. This deficiency has been shown to be an essential issue. For example, in a cross-sectional study, including editorial staff affiliated with LMICs was moderately associated with a higher proportion of published articles reporting research conducted in LMICs [44]. The author of this study suggests increased inclusion of editorial staff affiliated with LMICs. Finally, to confirm the deficiency of Arabic representation on editorial boards, we surveyed the editorial boards of ten prominent bioethics journals. While the number of editorial board members ranged from 16–50 (32.9+10.9), the number of Arab investigators on these editorial boards was three (see supplementary file 2 for the list of these bioethics journals).

## Conclusion

This study provides insight into why research publications from Arabic-speaking countries are Deficient. If Arab authors cannot learn the skills necessary to initiate research studies and lead publications, they will not become independent researchers, thereby perpetuating the dominance of scientists from high-income countries [5]. Identifying the challenges of Arab researchers in publishing original and meaningful research would guide programs for scientific research training at both undergraduate and postgraduate levels that are tailored and targeted toward Arab scholars’ needs. This information could be used by universities to develop the necessary training programs to enhance research productivity. Also, our results can be used by governments and international funders to raise and allocate funds to build collaborative relationships that improve research quality and scientific writing abilities.

## Figures and Tables

**Table 1:** Themes and sub-themes.

Quality of the research	Inadequate novel research ideas
	Lack of access to the literature
	Failure to critically analyze the literature
	Flawed research methodology
	
Inadequate research resources	Lack of funding
	Inadequate research facilities: laboratories, equipment, electronic databases
	Lack of statistical resources
	Inadequate library resources
	
Non-supportive institutional environment	Inadequate protected time
	No rewards for research activity
	Bureaucratic hurdles
	Perverse requirements for promotion
	Lack of mentoring
	Insufficient training
	Non-supportive research culture
	Deficient teamwork culture
	
Difficulties with Scientific Writing	Language barriers
	Poor scientific writing skills
	Inadequate publication services
	Plagiarism
	
Publication Submission Process	Choosing the right journal
	Authorship disputes
	Difficulties writing the cover letter
	Unhelpful and callous reviewers’ comments
	Responding to reviewers’ comments
	Occult discrimination
	
International collaborations	Enhance commitment toward research
	Improve quality of research
	Enhance scientific writing
	
Addressing the challenges	Enhance research culture
	Mentoring programs
	Funding
	Workshops
	Increase editorial board representation

## Data Availability

The datasets generated and/or analyzed during the current study are available in the OSF repository at: https://osf.io/nvcwj/?view_only=645dee3abfb44cffbe2d64c473cb8c01
